# The Nursing Service in the Dawn of Peace

**Published:** 1918-11-16

**Authors:** 


### THE NURSING SERVICE IN THE DAWN OF PEACE.

The sense of relief which the cessation of hostilities brings to the nursing sisterhood of the Empire cannot be put into words. There are two aspects of the war which have come more vividly under the notice of the doctors and nurses than of any others. One is the horrible, ceaseless stream of slaughter; the shattered lives and maimed minds; the inconceivable, incurable suffering. This which the public only realises spasmodically, when names of friends or acquaintances or friends' friends appear in the casualty list, is daily and hourly stamping itself on the brains of those who watch the convoys come in. That this should cease is the occasion of such intense relief as almost seems to throw the past into oblivion. It is as the rolling off of a burden which has many a time seemed too heavy to be borne another minute. For the moment it is joy such as can be tasted but once in a lifetime.

There is another aspect of this blessed armistice which is felt in overwhelming fashion in all military hospitals throughout the Empire, might we not say throughout the world? It is that recovery no longer means return to a furnace heated sevenfold hotter than before. That curse of warfare which poisoned the springs of healing has rolled away. The wounded are safe. They have finished their ordeal and the shadows no longer fall across the sisters as they note returning vigour in those whose lives have been spared, alas, "to fight another day."

This is not to say that the task is ended. Except perhaps in the clearing stations farthest up the line there can be no hint for many weeks to come of any relaxation. The arrears are so heavy, the shortage so tremendous, that the great machine must go whirling monotonously on its way even though the end be in sight. Lady Ampthill has been reminding the public that V.A.D. recruiting must still continue its course or the trained nurses may find themselves even yet overwhelmed under the mighty press of work. But the difference is that no one now minds. The body may be weary, but it is the mind which counts, and it is the mind which is now at ease. Once eliminate the factors which were for ever sapping hope, if not faith, in the women to whom our soldiers looked for healing and all is changed. If the war is ended our strength is doubled forthwith. We take a deep breath and go back to work which has now a new meaning and a new joy.

It is well; for the nurse's part in the war is but beginning. This is a halting-place between two epochs, and the one which lies ahead needs every ounce of hope and courage the human spirit can muster. The sterner aspects of the armistice have still to be reckoned with. The policing of many countries seems to have devolved upon the Allies, and beyond the weighty burdens of the next few months lie countless problems which nurses must help to solve, problems on which the well-being of mankind for ages to come will depend. The sick and the wounded, and of these there are millions, will need succour for months or years to come, some even for life. The wounds of France and Belgium, possibly of Poland and Russia, are waiting to be stanched; there is new work in the East; there are great needs in Africa; there is a heavy dip in the birth-rate to be countered, and its full force will not be felt for thirty years at least. If the influence of the war is not to blast this generation and the next the nurses must hear the call.

Had the nursing profession to face the labours of peace without organisation how desperate an enterprise must have seemed the task of the new era! But, as often happens to individuals in times of great stress, the nursing profession has found itself in the war. Slowly at first the impulse to combine made itself felt, has gathered force and touched with ardour those who alone can guide the forces of reform now making themselves felt. When once the nursing profession has consolidated itself under State protection in the College founded and endowed in the interests of the women who have been making history, the country will see an organisation strong enough to fulfil all the obligations, and they are beyond counting, which will be entailed on the nurses of the future.

Every deed of heroism achieved brings with it the call to further achievement. The nursing profession, united to-day as never before, pauses to look back and to look forward; and, conscious of strength proved in the sight of the world, tastes a joy which is eternal because it is of God.

